# Treatment of Burkitt lymphoma in real-world setting: findings on 104 consecutive cases diagnosed and treated in Kazakhstan over the last decade

**DOI:** 10.1007/s00277-026-06941-1

**Published:** 2026-03-19

**Authors:** Akmaral Jazyltayeva, Nazarii Shokun, Ayazhan Umutbayeva, Luana Conte, Raigul Ramazanova, Vadim Kemaikin, Azat Karabekov, Aidana Shalabay, Aigerim Koshkarbayeva, Nurbergen Kemelbekov, Madina Zhumabay, Fariza Shokubayeva, Yana Stepanihyna, Massimo Federico, Saule Gabbasova

**Affiliations:** 1 Center for Hematology and Bone Marrow Transplantation, Kazakh National Institute of Oncology and Radiology, Almaty, Kazakhstan; 2 Department of Experimental Medicine, Unisalento, Lecce, Italy; 3 Kazakh National Medical University, Almaty, Kazakhstan; 4 Department of Physics and Chemistry, University of Palermo, Palermo, Italy; 5 Center for Onco-hematology, National Scientific Research Center Astana, Astana, Kazakhstan; 6 Hematology Department, Jules Bordet Institute, Brussels, Belgium; 7 CHIMOMO Department, University of Modena and Reggio Emilia, Modena, Italy

**Keywords:** Burkitt lymphoma, Chemotherapy, Prognostic factors, Real-world data

## Abstract

We investigated treatment outcomes, relapse risk, and survival in 104 consecutive patients with non-endemic Burkitt lymphoma (BL) diagnosed and treated in Kazakhstan between 2013 and 2024 in a real-world setting. This was a retrospective, clinically based study analyzing baseline characteristics, treatment regimens, response to first-line therapy, prognostic factors, relapse, and survival. The median age was 26 years (range 2–80), with a male predominance (64%). All cases underwent MYC rearrangement assessment, and EBV status was available in 80 patients, with 28% testing positive. Curative-intent therapy was delivered to 95 patients (91%), including R-BFM (44%), R-EPOCH (23%), R-CODOX-M/R-IVAC (18%), and R-Hyper-CVAD (15%); no patient underwent autologous stem cell transplantation. After a median follow-up of 57 months, the 3-year overall survival (OS) and progression-free survival (PFS) were 57% and 56%, respectively. Patients younger than 18 years, all treated with R-BFM, had significantly superior outcomes compared with adults, with 3-year OS and PFS of 82% and 83%, respectively (*p* < 0.001). Among adults, patients treated with R-Hyper-CVAD or R-CODOX-M/R-IVAC had better OS than those treated with R-EPOCH (50% vs. 19%, *p* = 0.04). In univariate analysis, age ≥ 40 years, female sex, ECOG performance status ≥ 2, and platelet count < 150 g/dL were associated with inferior prognosis. In multivariate analysis, age ≥ 40 years remained the only independent adverse prognostic factor. The adapted Burkitt Lymphoma International Prognostic Index retained prognostic value. In conclusion, this large real-world study demonstrates excellent outcomes in pediatric patients treated with intensive immunochemotherapy but substantially inferior results in adults, particularly in refractory disease, highlighting a critical unmet clinical need in this population.

**Supplementary Information:**

The online version contains supplementary material available at 10.1007/s00277-026-06941-1.

## Introduction

Burkitt lymphoma (BL) is a rare and highly aggressive form of B-cell non-Hodgkin lymphoma, characterized by rapid tumor proliferation and typically associated with MYC translocations.1 BL manifests in three clinical forms: endemic, sporadic, and immunodeficiency-associated, with Epstein-Barr virus (EBV) playing a significant role particularly in endemic cases [1, 2]. Endemic BL occurs in regions with holoendemic Plasmodium falciparum malaria and is almost universally EBV-driven, whereas in non-malarial regions, including Kazakhstan, BL cases correspond to the sporadic form and are generally less frequently associated with EBV infection [2]. 

Approximately 95% of endemic BL cases are associated with EBV, underscoring its pivotal role in that epidemiological setting. However, not all BL cases are EBV-related, particularly in sporadic BL, where alternative pathogenetic mechanisms are implicated and remain incompletely understood [2]. EBV infection contributes to BL pathogenesis through mechanisms such as the downregulation of tumor suppressor genes like TP53 by EBV-miR-BART5-3p, thereby promoting uncontrolled cell proliferation [3]. The aggressive nature of BL necessitates timely diagnosis and treatment, as delays can lead to dismal outcomes. Intensive immunochemotherapy remains the cornerstone of treatment and has significantly improved survival rates [1, 4–7]. 

Despite these advances, geographically specific real-world data (RWD) on BL outcomes are scarce, particularly from regions without endemic malaria where BL is sporadic. RWD provide invaluable information on treatment successes and challenges in routine clinical practice, especially in settings where healthcare systems, patient demographics, and access to therapies differ from those in controlled clinical trials. By addressing this gap, the present study focuses on patients with sporadic BL living in Kazakhstan, a non-malarial region, and describes treatment patterns, outcomes, and the frequency of EBV-associated disease. These data highlight the importance of context-specific RWD in guiding treatment strategies and addressing the critical need for region- and setting-specific evidence.

## Patients and methods

The study was performed in accordance with the Declaration of Helsinki and was approved by the Local Ethics Committee of the Kazakh Institute of Oncology and Radiology.

This was a retrospective analysis of all consecutive patients with a diagnosis of BL who had been admitted to one of the 12 participating Oncological Cancer Centers in Kazakhstan between 2013 and 2024. Data were retrieved from electronic registry of cancer patients. Cases had adequate tissue biopsy specimens, and the diagnosis was based mostly on the 3rd (2008) and 4th (2017) edition of World Health Organization (WHO) classification.

EBV status in this cohort was assessed by quantitative PCR for plasma EBV DNA. This approach was selected because EBV-encoded RNA in situ hybridization (EBER-ISH) on tumor tissue was not routinely available during the study period across all participating centers, whereas plasma EBV PCR was increasingly implemented in routine diagnostics and follow-up. We acknowledge, however, that plasma EBV DNA is an imperfect surrogate for tumor EBV status in BL. EBV viremia may occur as a bystander phenomenon without reflecting tumor involvement, and conversely, EBV-positive tumor tissue may be associated with low or undetectable levels of circulating EBV DNA. Therefore, plasma EBV PCR cannot be assumed to be fully equivalent, in terms of sensitivity and specificity, to EBER-ISH on biopsy specimens for defining EBV-driven BL. In light of these limitations, the EBV data reported here should be interpreted strictly as a descriptive laboratory marker of circulating EBV DNA rather than definitive tumor EBV involvement, and PCR positivity was not equated with EBER-confirmed EBV-positive BL. Future prospective studies in this setting should incorporate standardized tissue-based EBV testing (e.g., EBER-ISH) to enable more precise assessment of the prevalence and clinical impact of EBV-associated BL in non-endemic regions such as Kazakhstan.

Clinical data, including demographics, baseline information on Eastern Cooperative Oncology Group (ECOG) performance status, serum LDH, Ann Arbor stage, extra nodal involvement, bone marrow involvement, central nervous system (CNS) involvement, the presence of bulky tumor more than 9 cm, HIV and EBV status were analyzed.

Bone marrow biopsy was performed at diagnosis in all patients as part of routine staging procedures.

### Age group definitions

For the purposes of analysis, patients were categorized according to age at diagnosis. Pediatric/adolescent patients were defined as those aged < 18 years, whereas adult patients were defined as those aged ≥ 18 years. In survival analyses, adult patients were further stratified into 18–60 years and > 60 years age groups, in accordance with the age categories presented in Fig. 2. Pediatric/adolescent patients (< 18 years) were treated exclusively with the R-BFM protocol, while adult patients (≥ 18 years) received adult-type regimens.

In our study, we applied a modified version of the Burkitt Lymphoma International Prognostic Index (BL-IPI) to evaluate its prognostic utility in a mixed-age real-world cohort that included both pediatric and adult patients. While the original BL-IPI was developed for adult populations and uses LDH > 3× the upper limit of normal (ULN) as a threshold, we adjusted the model to reflect the characteristics and data structure of our cohort. First, because a substantial proportion of our patients were younger than 18 years and were treated with pediatric-type protocols, we included these patients in the BL-IPI analysis to avoid excluding clinically relevant cases and to explore the performance of the index across the full age spectrum treated in routine practice in Kazakhstan. Second, given variability in historical laboratory reporting and incomplete documentation of exact LDH multiples above ULN in several centers, we used LDH > ULN (rather than > 3× ULN) as the operational cut-off to minimize misclassification and maximize the number of evaluable patients. On this basis, we defined four risk factors—age ≥ 40 years, ECOG performance status ≥ 2, LDH > ULN, and CNS involvement—and categorized patients into low (0), intermediate (1), and high (≥ 2) risk groups.

Patients were treated with R-BFM, R-EPOCH, R-Hyper-CVAD, or R-CODOX-M / R-IVAC. For patients receiving R-BFM the treatment plan consisted of 3 blocks, block A (with ifosfamide, vincristine, methotrexate, etoposide, and low-dose cytarabine), block B (containing vincristine, cyclophosphamide, methotrexate, and doxorubicin), and block C (with vindesine, methotrexate, etoposide, and high-dose cytarabine). Each block was repeated twice, every 28 days. Rituximab was given at day 1 of each block at the standard dose of 375 mg/m^2^, regardless of age. Intrathecal prophylaxis (ITP) was given once per each block. R-EPOCH was delivered as infusion of intravenous rituximab 375 mg/m^2^/day, prior to each chemotherapy, and 96-h continuous infusion of etoposide (50 mg/m^2^/day), doxorubicin (10 mg/m^2^/day), and vincristine (0.4 mg/m^2^/day), plus oral prednisone (60 mg/m^2^/day) from day 1 to day 5 and intravenous cyclophosphamide 375 mg/m^2^ on day 5, every 21 days. R-Hyper-CVAD consisted from 8 cycles. Cycles 1, 3, 5, 7 included cyclophosphamide 300 mg/m^2^ IV every 12 h for six doses d 1–3, doxorubicin 50 mg/m^2^ IVI over 48 h d4, vincristine 2 mg/m^2^ IV (maximum 2 mg) d4 & d11, dexamethasone 40 mg IV or PO d1-5 and d11-14. Cycles 2, 4, 6, 8 included methotrexate 200 mg/m^2^ IV bolus d1, methotrexate 800 mg/m^2^ continuous IVI over 24 h d1, cytarabine 3,000 mg/m^2^ IV over 2 hrs every 12 h for four doses d 2–3. R-CODOX-M included rituximab 375 mg/m^2^ on d 1 and 9, cyclophosphamide 800 mg/m^2^ on d 1, cyclophosphamide 200 mg/m^2^ on d 2–5, vincristine 1.5 mg/m^2^ on days 1 and 8, doxorubicin 40 mg/m^2^ on d 1, and methotrexate 3000 mg/m^2^ on d 10. R-IVAC consist of rituximab 375 mg/m^2^ on d 3 and 7, ifosfamide 1500 mg/m^2^ on d 1–5, etoposide 60 mg/m^2^ on d 1–5, and cytarabin 2000 mg/m^2^ on d 1, 2).

The principal end point of this study was OS, measured from the date of diagnosis until death from any cause or the date of the last known contact (in the year 2024) for living patients. Additional end point was PFS measured from the date of diagnosis until to date of relapse after previous complete remission or progression after reaching partial remission (50% decrease and resolution of B symptoms and no new lesions); progressive disease (50% increase from nadir of any previous partial remission lesions or appearance of new lesions) on computed tomography scan measurements during treatment; or death from any cause, whichever occurred first. PFS was assessed exclusively in relation to first-line therapy; subsequent lines of treatment did not influence the PFS endpoint.

Due to the retrospective nature of the study and the lack of structured toxicity reporting in the electronic cancer registry, data on treatment-related adverse events and therapy-associated complications could not be systematically retrieved or reliably analyzed.

### Imaging and response assessment

Baseline staging and response assessment were performed using contrast-enhanced computed tomography (CT) in all participating centers. Positron emission tomography combined with CT (PET/CT) was not routinely available during the study period and was therefore not systematically used. Disease response was evaluated according to radiological criteria based on changes in tumor burden on CT imaging, together with clinical assessment, in routine clinical practice. Due to the retrospective nature of the study and heterogeneity in imaging availability over time and across centers, standardized response criteria such as the Lugano classification could not be uniformly applied.

The study was conducted in compliance with the Declaration of Helsinki and approved by the Local Ethic Committee of the Kazakh Institute of Oncology and Radiology.

Survival curves were calculated with the Kaplan-Meier method, and time-to-event distributions were compared with the log-rank test (univariate regression). Cox models were used to investigate the association between survival outcomes and covariates with hazard ratios (HRs) with 95% Cis used as summary measure. Statistical analyses were performed using SPSS (version 29.0).

## Results

A total of 104 patients with non-endemic classical BL from 12 different Centers in Kazakhstan were retrieved and analyzed. Baseline characteristics are shown in Table 1. Briefly, median age was 26 years (range 2–80), with male predominance (67 patients, 64%). 97% of patients had MYC rearrangement on pathology review. EBV status was assessed in 80 patients and was positive in 22 (28%).

**Table 1:** Baseline characteristics of patients with Burkitt Lymphoma

Variable	*N* – 104 (%)
Age, median (range)	26.5 (2–80)
Age ≥ 40, n (%)	37 (35.6)
Gender, n (%)	
Male	67 (64.4)
HIV-positive, n (%)	7 (6.7)
PS ECOG ≥ 2, n (%)	64 (61.6)
Stage 3 or 4, n (%)	65 (62.5)
CNS involvement, n (%)	8 (7.7)
LDH ≥ ULN, n (%)	52 (50)
Bone marrow involvement	0
B symptoms	61 (58.7)
Hemoglobin < 12 g/dL	82 (78.8%)
Platelets < 150 g/dL	5 (4.8)
Bulky	45 (43.3)
*MYC* rearrangement	103 (99)
Extranodal sites	25 (24.0)
IPI 3–5 (*N* = 100)	41 (39.5)
BL-IPI High Risk (≥ 2 factors)	53 (51.0)
EBV positive (*N* = 80 assessed)	22 (27.5)
First-line regimen, N	**95**
BFM, n (%)	42 (44.2)
R-Codox-IVAC, n (%)	17 (17.9)
R-EPOCH, n (%)	22 (23.2)
R-Hyper CVAD, n (%)	14 (14.7)
Follow-up, median	57.1 (50.1–64)
OS at 3 years, 95% CI	57.2 (51-69.4)
PFS at 3 years, 95% CI	56.2 (46.2–66.5)

*BFM* Berlin-Frankfurt-Münster protocol, *BL-IPI* Burkitt Lymphoma International Prognostic Index, *CNS* Central Nervous System, *EBV* Epstein-Barr Virus, *ECOG* Eastern Cooperative Oncology Group, *IPI* International Prognostic Index, *LDH* Lactate Dehydrogenase, *MYC* v-myc avian myelocytomatosis viral oncogene homolog, *R-Codox-IVAC* Rituximab, Cyclophosphamide, Doxorubicin, Vincristine + Ifosfamide, Etoposide, and high-dose Cytarabine; *R-EPOCH* Rituximab, Etoposide, Prednisone, Vincristine, Cyclophosphamide, Doxorubicin, *R-Hyper CVAD* Rituximab, Hyperfractionated Cyclophosphamide, Vincristine, Doxorubicin, Dexamethasone, High-dose Methotrexate, Cytarabine

Sixty-five patients (62%) had advanced Ann Arbor stage; 61 (59%) presented with B symptoms, 52 (50%) with elevated LDH, 25 (24%) with more than one extra nodal site. Seven (7%) patients were HIV positive, 8 (8%) patients had CNS involvement at diagnosis, and no one had bone marrow involvement for all evaluated cases.

Nine patients did not receive any anti-lymphoma treatment and died soon after diagnosis. A summary of these patients’ history is reported in Supplementary Table 1. Two had HIV associated BL: one declined both chemotherapy and antiretroviral therapy and one untreated due to the poor performance status. Reasons for treatment omission in the remaining seven untreated patients were poor performance status (ECOG 3–4), severe comorbidities (e.g., heart failure, chronic kidney disease, hepatic dysfunction), and rapid clinical deterioration. Among the 8 patients deemed unfit for treatment, survival time ranged from 2 to 21 days, with a median of 10 days, highlighting the aggressive nature and poor prognosis in untreated BL.

In the remaining 95 patients the adopted chemotherapy regimens were R-BFM (42 patients, 44%), R-EPOCH (22 patients, 23%), R-CODOX-M / R-IVAC (17 patients, 18%) and R-Hyper-CVAD (14 patients, 15%). R-BFM was used in pediatric and adolescent patients only, whilst the other regimens were used in adults. Due to the similarity, we merged the intensive regimens R-CODOX-M / R-IVAC and R-Hyper-CVAD in a single group. Clinical characteristics/differences among the three treatment groups are shown in Supplementary Table 2. No patient was intensified with ASCT. Three patients were lost to follow-up before response assessment and have been excluded from further analyses.

**Fig. 1:**
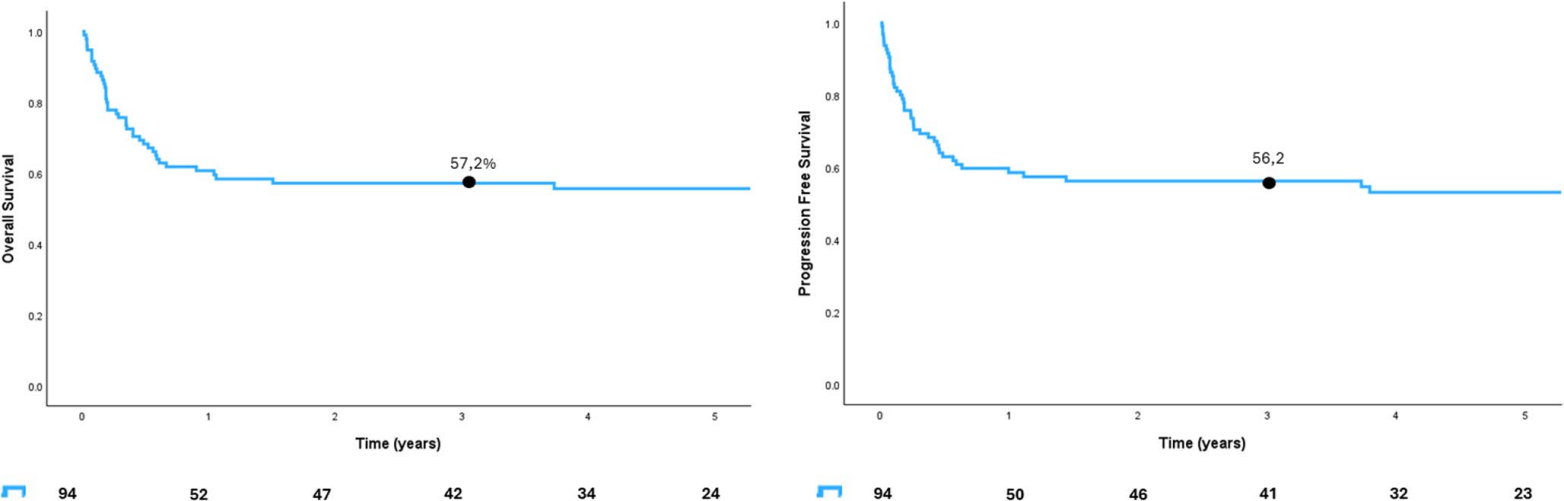
Kaplan–Meier survival curves for Overall Survival (OS) and Progression-Free Survival (PFS). The left panel shows the OS curve with a 57% survival rate at 3 years. The right panel displays the PFS curve with a 56% survival rate at 3 years. The tables below each plot indicate the number of patients at risk and the corresponding survival percentages over time

Fifty-one (54%) out of 95 patients treated with curative intent and assessed for response to initial therapy, achieved Complete Remission (CR), 9 Partial Remission (PR) (9%) and 35 No Response (NR) (37%), with an overall response rate (ORR) of 64%. Patients receiving R-BFM had the highest ORR (47 patients, 92%) compared to other regimen (21 patients, 40%) (Table 2). After a median follow-up of 57 months (95% CI 50.1% – 64%) for living patients, the 3-year OS and PFS were 57% (95% CI, 51-69.4%) and 56% (95% CI, 42–66%), respectively (Fig. 1a and b**).**

**Table 2:** Response to initial therapy

Response / Therapy	*R*-BFM	*R*-Codox-IVAC / *R*-Hyper CVAD	*R*-EPOCH	Total
CR	34 (80.9%)	14 (45.1%)	3 (13.6%)	51 (53.7%)
PR	4 (9.5%)	3 (9.7%)	2 (9.1%)	9 (9.5%)
PD	4 (9.5%)	14 (45.2%)	17 (77.3%)	35 (36.8%)
Total	42 (100%)	31 (100%)	22 (100%)	95 (100%)

*BFM* Berlin-Frankfurt-Münster protocol, *CR* Complete Response, *PD* Progressive Disease, *PR* Partial Response, *R-Codox-IVAC* Rituximab, Cyclophosphamide, Doxorubicin, Vincristine + Ifosfamide, Etoposide, and high-dose Cytarabine, *R-EPOCH* Rituximab, Etoposide, Prednisone, Vincristine, Cyclophosphamide, Doxorubicin; *R-Hyper CVAD* Rituximab, Hyperfractionated Cyclophosphamide, Vincristine, Doxorubicin, Dexamethasone, High-dose Methotrexate, Cytarabine

3-year OS was 92%, 89% and 0% for patients who achieved CR, PR or NR (*p* < 0.001) (Supplementary Fig. 1a). 3-year PFS was 90%, 89% and 0% for patients who achieved CR, PR, NR respectively (*p* < 0.001) (Supplementary Fig. 1b).

Among HIV-positive patients, 5/7 received lymphoma-directed therapy (R-CODOX-M/R-IVAC, *n* = 4; R-EPOCH, *n* = 1). Of these, 4/5 developed PD and died, while 1/5 achieved PR and was alive at last follow-up.

**Fig. 2:**
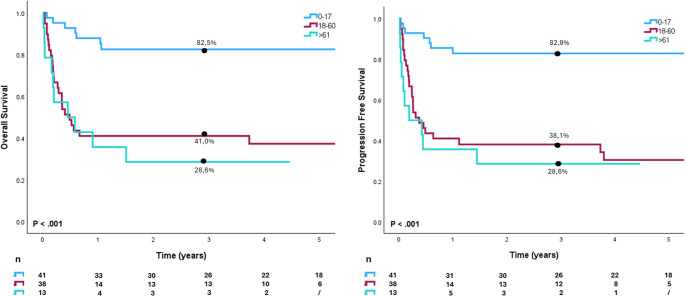
Kaplan-Meier survival curves illustrating Overall and Progression-Free Survival stratified by age group. Panel (**A**) Overall Survival (OS). Patients aged 0–17 years (blue) had significantly higher 3-year OS (82%) compared to those aged 18–60 years (red, 41%) and those over 61 years (cyan, 29%) (*P* < 0.001). Panel (B) Progression-Free Survival (PFS). Similarly, the 3-year PFS was highest in the 0–17 age group (83%), while adults aged 18–60 and > 61 years showed lower PFS rates (38% and 28.8%, respectively) (*P* < 0.001). These curves reflect age-stratified outcomes and are not intended as direct comparisons of frontline treatment regimens across pediatric and adult populations

The group of patients aged less than 18 years (all of them treated with R-BFM) had a significantly better outcome compared with those aged 18–60 and > 60 years, both in terms of OS (*p* < 0.001) and PFS (*p* < 0.001) (Fig. 2a and b). These analyses are age-stratified and reflect outcomes within clinically defined treatment groups; they are not intended as direct comparisons of different frontline regimens across pediatric and adult populations.

**Fig. 3:**
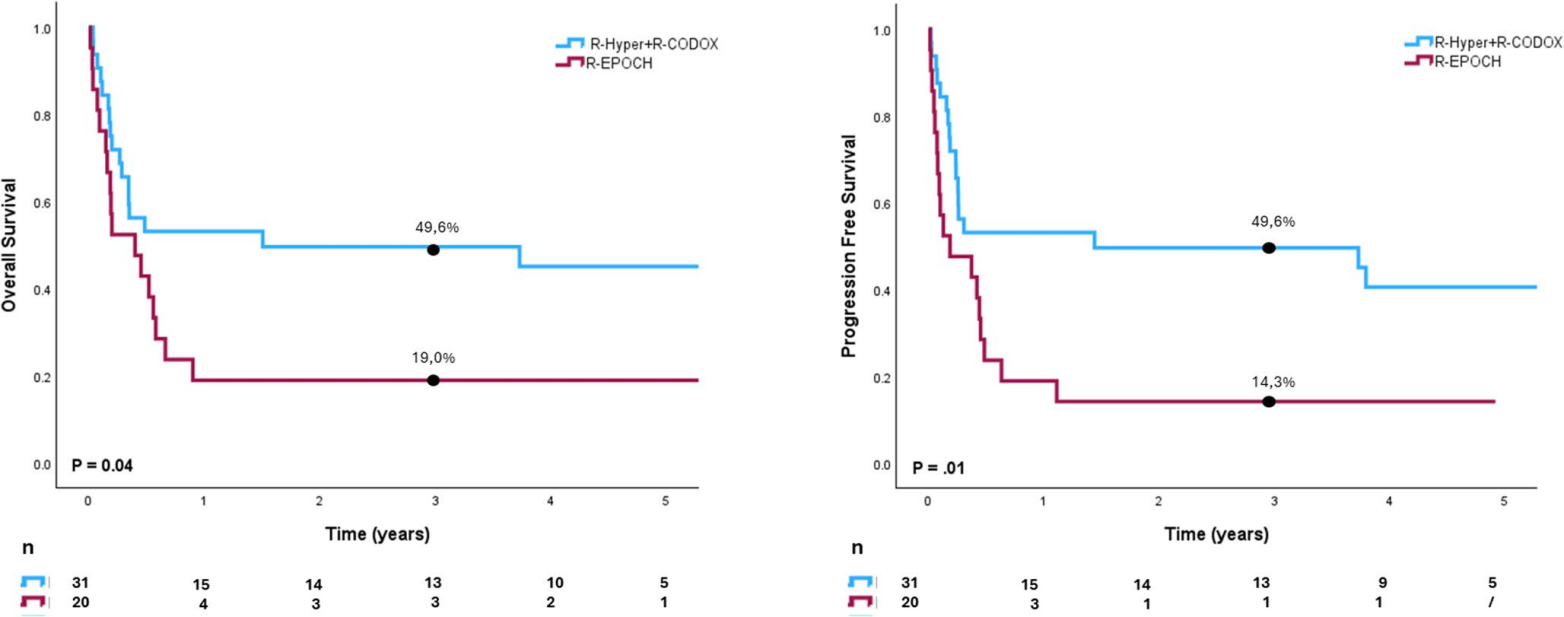
Kaplan-Meier survival curves comparing treatment outcomes between patients treated with R-Hyper CVAD + R-CODOX (blue line) and those treated with R-EPOCH (red line) in our cohort. Panel **A**) Overall survival (OS). Patients treated with R-Hyper CVAD + R-CODOX had significantly better OS (50%) compared to those receiving R-EPOCH (19%) at 3 years (P = .04).Panel **B**) Progression-free survival (PFS). Similarly, R-Hyper CVAD + R-CODOX was associated with improved PFS (45%) versus R-EPOCH (14%) at 3 years (P = .01).

Interestingly, in adult patients, those treated with R-Hyper-CVAD or R-CODOX-M / R-IVAC had a better outcome compared with those treated with R-EPOCH, both in terms of OS(*p* < 0.001) and PFS (*P* = 0.01) (Fig. 3a and b).

Male sex was associated with better outcomes (HR 0.54, 95% CI 0.29–0.99, *p* = 0.048).

Patients with an ECOG score of 2 or higher accounted for 56 (59%) cases. Among them, the 3-year OS and PFS were 47% and 46%, respectively, compared with 71% and 71% observed in patients with an ECOG score of 0–1 (p-value < 0.01). Furthermore, 61% (17 out of 26) patients with ECOG 3–4 did not respond to first-line therapy, and 18 of these patients died within 12 months since diagnosis.

In the present series LDH levels were not significantly associated with survival outcomes. Specifically, patients with normal LDH levels had a 3-year OS of 63% and a PFS of 52%, compared with 52% OS and 60% PFS in patients with LDH levels above normal range.

In patients with B-symptoms 3-year OS was 53% and the PFS was 52%, whereas in patients without B symptoms, the OS and PFS were 62% and 62%, respectively.

Patients (60%) diagnosed with stage III–IV exhibited 51% 3-year OS compared with 66% for patients in earlier stages, and the difference was not statistically significant. However, when isolating stage IV cases (26 patients, 27%), the 3‐year OS dropped to 42% with a PFS of 38%, which was significantly lower than the OS (63%) and PFS (63%) observed in patients with stage I-III (*p* < 0.05).

Bulky disease was present in 45 patients (43%). These patients had a 3-year OS of 47% and a PFS of 45%, compared with 65% OS and 66% PFS in patients without bulky disease, though these differences were not statistically significant.

EBV status was not significantly associated with outcome; patients with positive EBV status exhibited a 3-year OS of 55% and a PFS of 55%. In contrast, the EBV‐negative group showed 3‐year OS and PFS rates of 42% and 44%, respectively.

In the multivariable model, only age ≥ 40 years remained independently associated with inferior PFS (HR 1.944, 95% CI 1.02–3.71, *p* = 0.04), whereas ECOG ≥ 2 showed a non-significant trend (Table 3).

**Table 3:** Univariable and Multivariable Models for association between prognostic variables and Progression-Free Survival (95 cases)

Variable		Univariable			Multivariable	
	HR	95% CI	*P*	HR	95% CI	*P*
Age ≥ 40	2.477	1.357 to 4.519	0.003	1.944	1.020 to 3.706	0.04
Male	1.969	1.080 to 3.588	0.0271	1.343	0.697 to 2.588	0.38
ECOG ≥ 2	2.355	1.183 to 4.686	0.015	1.804	0.860 to 3.785	0.12
Stage ≥ 3	1.840	0.958 to 3.535	0.067			
B symptoms	1.375	0.740 to 2.553	0.314			
CNS involvement	0.864	0.599 to 1.247	0.434			
LDH ≥ ULN	0.836	0.459 to 1.522	0.558			
Hemoglobin < 12 g/dL	1.401	0.690 to 2.847	0.351			
Platelets < 150 g/dL	0.267	0.093 to 0.764	0.014	0.528	0.174 to 1.603	0.26
EBV positive	0.965	0.674 to 1.380	0.844			
Bulky	1.706	0.933 to 3.120	0.08			
IPI ≥ 2	4.809	2.551–9.066	< 0.001			
BL IPI ≥ 2	1.806	1.124 to 2.902	0.015			

*BL IPI* Burkitt Lymphoma International Prognostic Index, *CNS* Central Nervous System, *ECOG* Eastern Cooperative Oncology Group, *EBV* Epstein-Barr Virus, *HR* Hazard Ratio, *IPI* International Prognostic Index, *LDH* Lactate Dehydrogenase, *ULN* Upper Limit of Normal

Risk categorization based on the number of adverse prognostic factors—age ≥ 40 years, ECOG performance status ≥ 2, LDH > ULN, and CNS involvement—showed that 17 patients (18%) had no risk factors (low risk), 33 patients (36%) had one factor (intermediate risk), and 42 patients (47%) had two or more (high risk) (Supplementary Fig. 2).

**Fig. 4:**
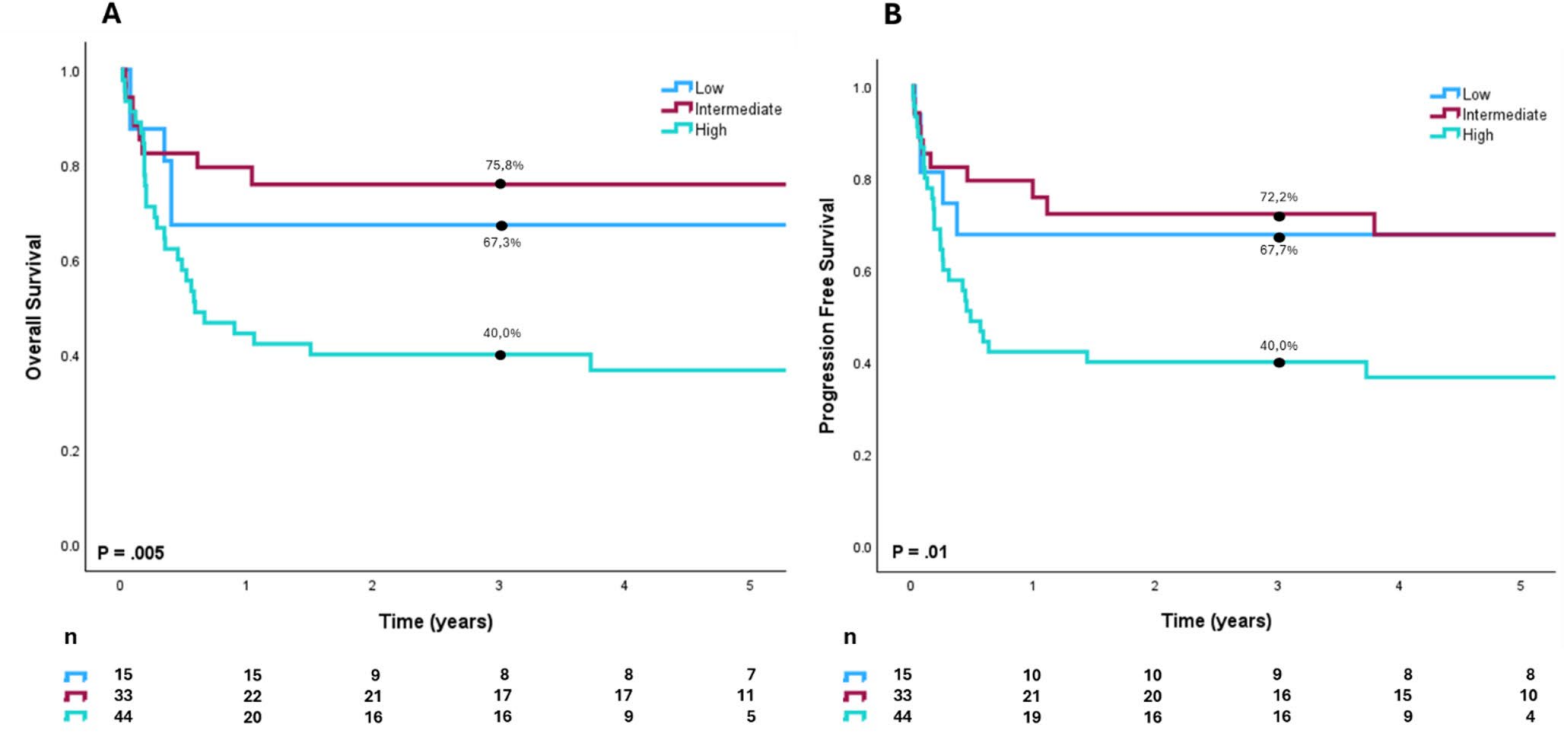
Performance of the BL-IPI. Panel (**A**) Overall Survival according to BL-IPI risk group; (**B**) Progression Free Survival according to BL-IPI risk group. Log-rank p-values are calculated to compare groups and for overall analysis. Three-year Overall Survival and Progression Free Survival are listed. BL-IPI: Burkitt lymphoma International Prognostic Index.

%) The adapted BL-IPI effectively discriminated outcomes in our cohort. As shown in Fig. 4, the 3-year OS rates for patients with low, intermediate, and high BL-IPI scores were 67%, 76%, and 40%, respectively (*p* = 0.005). Similarly, PFS at 3 years was 68% for low-risk, 72% for intermediate-risk, and 40% for high-risk patients (*p* = 0.01). These findings support the prognostic utility of the BL-IPI in our population, with high-risk patients showing substantially inferior outcomes.

Thirty-nine patients received second-line therapy: 26 (26%) were treated with curative intent, whilst 13 (14%) were referred to best supportive care. The three most frequent second-line regimens were: R-Hyper-CVAD, R-DA-EPOCH, and CHOP-like in 10 (11%), 5 (5%), and 4 (4%) patients, respectively. Only 4 (4%) patients achieved a second CR. The median time from diagnosis to disease progression or relapse was 76 days (0.2–45.6 months) and the median time from diagnosis to death was 20 days (0.1–10.3 months) (Table 4).

**Table 4:** Characteristics of Relapse/Refractory (R/R) patients

Characteristics	Count (*N*, (%)
**Age**,** median (range)**	40 (5–79)
Age ≥ 40	20 (51.28%)
Male	21 (53.85%)
ECOG ≥ 2	29 (74.36%)
LDH > ULN	25 (64.1%)
B-symptoms	23 (58.97%)
Bulky	21 (53.85%)
Hgb < 12.0	31 (79.4%)
PLT < 150	4 (10.26%)
**EBV**	
Positive	21 (53.85%)
Negative	11 (28.21%)
NA	7 (17.95%)
**Salvage therapy**	
CHOP-like	4 (10.26%)
LMB-like regimen	3 (7.69%)
BFM/GMALL-like regimen	1 (2.56%)
Other	1 (2.56%)
Palliative care only (e.g. corticosteroids)	12 (30.77%)
R-DA-EPOCH	5 (12.82%)
R-Hyper CVAD	10 (25.64%)
R-CODOX-M / R-IVAC	1 (2.56%)
**Response**	
CR	2 (5.13%)
PR	2 (5.13%)
SD	25 (64.1%)
PD	8 (20.51%)

*BFM* Berlin-Frankfurt-Münster protocol, *CHOP* Cyclophosphamide, Doxorubicin, Vincristine, Prednisone, *CR* Complete Remission, *EBV* Epstein-Barr Virus, *ECOG* Eastern Cooperative Oncology Group, *GMALL* German Multicenter Study Group for Adult Acute Lymphoblastic Leukemia, *Hgb* Hemoglobin, *LDH* Lactate Dehydrogenase, *LMB* Lymphomes Malins B, *NA* Not Available, *PD* Progressive Disease, *PLT* Platelets, *PR* Partial Remission, *R-DA-EPOCH* Rituximab, Dose-Adjusted Etoposide, Prednisone, Vincristine, Cyclophosphamide, Doxorubicin,* R-Hyper CVAD* Rituximab, Hyperfractionated Cyclophosphamide, Vincristine, Doxorubicin, Dexamethasone, High-dose Methotrexate, *Cytarabine* R-CODOX-M, Rituximab, Cyclophosphamide, Vincristine, Doxorubicin, high-dose Methotrexate; *R-IVAC* Rituximab, Ifosfamide, Etoposide, high-dose Cytarabine, *R/R* Relapsed/Refractory, *SD* Stable Disease

In our retrospective analysis, 50(48%) patients died, 41 (82%) due to the lymphoma progression or relapse, 2 (4%) from infection, and 5 (10%) from other reasons that weren’t captured by the physician.

## Discussion

In this real-world data setting of 104 consecutive BL cases diagnosed and treated in Kazakhstan over the last decade, we recorded an overall response rate of 63%, 3-year OS of 57%,and PFS of 56% that appears slightly inferior to that recently reported in other studies [8–10]. However, the differences were evident only in adults because pediatric and adolescent patients exhibited an excellent outcome, in line with a recent report by Vural et al. [11] on 42 children patients treated with modified BFM protocol [11]. Importantly, the superior outcomes observed in pediatric and adolescent patients should be interpreted in the context of younger age, disease biology, and the use of pediatric-intensive therapy, and do not represent a head-to-head comparison of treatment regimens across age groups.

Our pediatric/adolescent outcomes (3‑year OS 82% and PFS 83% with R‑BFM) are consistent with contemporary pediatric Burkitt lymphoma series from high‑income settings. In the Turkish multicenter experience of high‑risk pediatric BL treated with a modified BFM regimen, 10‑year OS and EFS were 90% and 88%, respectively, with no deaths from refractory disease, only from treatment‑related toxicity [11]. Similarly, European cooperative group studies using BFM‑ or LMB‑based protocols have reported 5‑ to 10‑year OS/EFS in the 80–90% range for children and adolescents, even in predominantly stage III/IV cohorts. Large North American Children’s Oncology Group trials employing LMB‑inspired intensive short‑course regimens have also achieved long‑term EFS and OS ≥ 85–90% in pediatric BL [12, 13]. Thus, our pediatric figures, while numerically slightly lower, fall within the confidence intervals and are broadly aligned with high‑income pediatric BL benchmarks.\n\nIn contrast, pediatric BL series from low‑ and middle‑income countries continue to show substantially poorer survival despite protocolized therapy. In Malawi, a prospective study of children ≤ 18 years treated with six cycles of CHOP reported an 18‑month OS of only 29%. More recently, implementation of a national cyclophosphamide–vincristine–methotrexate–based regimen in Tanzania yielded a 1‑year OS of ~ 39% and EFS of ~ 30%, with high rates of advanced stage and treatment abandonment [14]. Earlier Malawian cohorts treated largely with cyclophosphamide monotherapy reported long‑term survival around 60% only in limited‑stage facial disease, with much poorer outcomes for abdominal or disseminated presentations [15]. When viewed against this broader pediatric BL literature, the 3‑year OS/PFS of > 80% in our R‑BFM–treated children and adolescents compare favorably to most LMIC series and approach the results reported in European and North American cooperative studies. These observations underscore that, even in a middle‑income setting with constrained resources, application of intensive BFM‑type pediatric protocols and structured supportive care can achieve outcomes approaching those of high‑income pediatric programs, in sharp contrast to the persistently poor survival reported for pediatric BL in many sub‑Saharan African cohorts. At the same time, they highlight the striking divergence between our pediatric and adult results, reinforcing the need to adapt similarly intensive, risk‑stratified, and well‑supported strategies for adult BL where feasible.

Zhu et al. [8] reported excellent outcomes in adults treated with intensive regimens such as CODOX-M/IVAC (with or without rituximab), with a 5-year OS of approximately 77% and PFS of 75% in a large population-based study from British Columbia. Similarly, Jakobsen et al. [9] demonstrated a 2-year OS of 84% and event-free survival (EFS) of 80% in an international multicenter series of 264 real-world BL patients [8, 9]. In contrast, our adult patients exhibited a 3-year OS of only 60%, suggesting that differences in patient demographics, supportive care infrastructure, or delays in diagnosis and treatment initiation may contribute to inferior outcomes in our setting.

A key factor potentially explaining this disparity is the difference in baseline performance status. In the Zhu dataset, only 38% of patients had an ECOG score > 2, and in the Jakobsen series, this figure was 31% [8]. By comparison, in our cohort, 40 out of 63 adult patients (63%) had an ECOG score of 2 or higher at diagnosis, reflecting a substantially more debilitated patient population and likely contributing to the poorer outcomes observed.

Further comparisons can be drawn with earlier pivotal studies. The original study by Magrath et al. using a pediatric-based intensive regimen in BL yielded a 2-year event-free survival as high as 92% in children and young adults [10]. More recently, Broccoli et al. reported 10 years OS and PFS of 83.7% and 76% respectively, in a group of 50 patients, 80% of whom were younger than 60 years, with no significant difference in terms of OS and PFS compared to the older group [16]. In our analyses, only patients 18 years or younger received high high-intensity R-BFM regimen with 82% 3-year OS and PFS.

Subsequent trials utilizing regimens such as Hyper-CVAD plus rituximab (Thomas et al., [17]) and modified CODOX-M/IVAC protocols (Mead et al., [18]) have confirmed the potential for high cure rates when optimal dose intensity is maintained [17, 18]. Moreover, phase II trials of DA-EPOCH-R (Dunleavy et al., [19]) have also reported remarkable outcomes in selected cohorts of adult patients [19]. Recently, Chamuleau et al. reported the final results of a multicentre, randomised trial comparing CODOX-M/IVAC with DA-EPOCH-R, and 2-year OS and PFS were not significantly different. For the DA-EPOCH-R group, it was 75% and 70% respectively [20].

In our study, adult patients treated with R-Hyper-CVAD or R-CODOX-M/R-IVAC achieved significantly better survival compared with those receiving R-EPOCH, suggesting that regimen intensity and patient selection are critical determinants of outcome. However, the median age in the R-EPOCH group was 60 years compared to 38 in the groups of R-Hyper-CVAD or R-CODOX-M/R-IVAC, and 15 of 21 of those patients had ECOG 2–4, which could bias our result.

It is important to note that while our pediatric and adolescent subgroup (all treated with R-BFM) performed well—consistent with historical data in BL—the overall lower response rate and survival in adults may be multifactorial. Factors such as the higher prevalence of advanced-stage disease, elevated LDH, and the potential for treatment delays in a resource-variable healthcare environment likely play a role. In addition, differences in supportive care measures and toxicity management may further account for the observed discrepancies.

In our study, we applied a modified version of the Burkitt Lymphoma International Prognostic Index (BL-IPI) to evaluate its prognostic utility in a mixed-age real-world cohort that included both pediatric and adult patients [21–23]. While the original BL-IPI was developed for adult populations and used LDH > 3× ULN as a threshold, we adjusted the model by including patients under 18 years and using LDH > ULN as the cut-off. Despite these modifications, the adapted BL-IPI retained its discriminatory power: the high-risk group (≥ 2 risk factors—age ≥ 40, ECOG ≥ 2, LDH > ULN, CNS involvement) had a 3-year OS of 40% and PFS of 40%, compared with 59% and 53% in the original derivation cohort. Likewise, the low-risk group in our cohort showed favorable outcomes, with a 3-year OS of 67% and PFS of 68%, compared with 96% and 92% reported in the original study. These findings underscore that the BL-IPI, even when slightly adapted, remains a reliable and practical tool for risk stratification in real-world clinical settings. It effectively identifies high-risk patients with poor outcomes and supports its use in routine practice, including in diverse healthcare environments and mixed-age populations.

Although Rituximab is becoming widely available globally, relapse or progression in Burkitt lymphoma remains almost universally fatal. A real-world retrospective study by Oosten et al. [24] reported that 21 out of 22 patients with relapsed or refractory disease died within one month of progression [24]. Although multiple retrospective and prospective studies have explored salvage approaches in relapsed or refractory Burkitt lymphoma, no universally accepted curative standard has yet emerged, and outcomes remain uniformly poor. Consistent with these findings, our study also demonstrated poor outcomes, with only 2 of 38 patients alive at the time of last follow-up, regardless of the salvage chemotherapy regimen used.

Our findings highlight the need for region-specific strategies to improve outcomes in BL. Although intensive immunochemotherapy remains the backbone of BL treatment, our data suggest that adaptations—whether in treatment regimen, supportive care optimization, or early intervention—are warranted for adult patients in Kazakhstan. Furthermore, our study reinforces the utility of real-world data in providing insight into treatment effectiveness outside controlled clinical trials, especially in regions where healthcare delivery and patient characteristics differ from those in North America or Europe.

Several limitations of this study should be acknowledged. First, due to the retrospective nature of the analysis and the absence of comprehensive and standardized documentation of treatment-related adverse events in the electronic cancer registry, therapy-associated toxicities and their contribution to mortality could not be reliably assessed. Second, cause-of-death attribution was entirely dependent on physician documentation and, in a minority of cases, remained indeterminate, which may have led to misclassification. Third, baseline staging and response assessment were performed according to routine clinical practice using contrast-enhanced computed tomography, while positron emission tomography/computed tomography was not systematically available; moreover, standardized response criteria could not be uniformly applied, potentially affecting staging accuracy and response evaluation. Fourth, heterogeneity in treatment approaches across centers and over time, as well as the inclusion of both pediatric and adult patients receiving different therapeutic strategies, may have introduced additional variability. Finally, although this represents the largest multicenter Burkitt lymphoma series from Kazakhstan to date, the sample size remains limited for certain subgroup analyses. These limitations are inherent to real-world retrospective studies and highlight the urgent need for prospective national registries with standardized data capture, including systematic toxicity, imaging, and outcome reporting.

In conclusion, while pediatric BL patients in our series achieve outcomes comparable to international benchmarks, the adult population shows a significant gap. Future prospective studies are needed to refine treatment protocols, enhance supportive care, and ultimately improve survival for adults with BL in Kazakhstan. These efforts will be crucial to bridge the gap between our real-world outcomes and those reported in international series such as Zhu et al. [8], Jakobsen et al. [9], and other seminal studies in BL [8, 9].

## Supplementary Information

Below is the link to the electronic supplementary material.

Supplementary Material 1.

## Data Availability

The datasets generated and/or analyzed during the current study are not publicly available due to institutional and data-protection restrictions, but are available from the corresponding author on reasonable request.
