# Supplementary material for: Treatment of Burkitt lymphoma in real-world setting: findings on 104 consecutive cases diagnosed and treated in Kazakhstan over the last decade
Source: Ann Hematol. 2026 Mar 19;105(4):189. doi: 10.1007/s00277-026-06941-1 (PMC12999861; doi:10.1007/s00277-026-06941-1)
Supplement: Supplementary file 1 — Supplementary Material 1. [file 277_2026_6941_MOESM1_ESM.docx]

|  | | | **SUPPLEMENTARY TABLE 1.** Patient history of nine patients who received no treatments. | | | | | | | | |
| --- | --- | --- | --- | --- | --- | --- | --- | --- | --- | --- | --- |
|  | **Patient id** | **Date of diagnosis** | | **Age at diagnosis** | **Stage** | **Comorbidities** | **HIV status** | **Reason for no treatment** | **Date of death** | **Cause of death** | **Notes** |
| 1 | **BL03** | 29.01.2019 | | 51 | IVB | Chronic heart failure, chronic kidney disease stage 3 | No | Poor PS, rapid deterioration, multiple comorbidities | 22.02.2019 | Massive upper gastrointestinal hemorrhage secondary to gastrointestinal involvement by advanced-stage lymphoma, complicated by peritoneal carcinomatosis and multiorgan failure. | Patient was admitted with extensive lymphomatous lesions in the retroperitoneum, iliac and inguinal regions, accompanied by peritoneal carcinomatosis and ascites. He developed bleeding from the upper gastrointestinal tract, resulting in hemodynamic instability. Due to progressive disease, concomitant CKD and CKD, and rapid clinical deterioration, he was ineligible for chemotherapy and died within 1 month of diagnosis. |
| 2 | **BL05** | 02.02.2015 | | 50 | IVB | Chronic obstructive pulmonary disease  Chronic kidney disease, stage 3a  Hepatic dysfunction | No | Poor PS (ECOG 3), advanced stage, comorbidities | 10.02.2015 | Progressive multiorgan failure secondary to disseminated lymphoma with peritoneal carcinomatosis | Patient was diagnosed on 02.02.2015 and died 8 days later on 10.02.2015. Disease involvement included peritoneal carcinomatosis with ascites, retroperitoneal, iliac, and inguinal lymphadenopathy. Supportive and palliative care were provided. |
| 2 | **BL16** | 13.10.2020 | | 44 | IIIB | HIV infection (not on a ARV therapy)  Chronic kidney disease  Liver dysfunction | Yes | Poor PS (ECOG 3), rapid deterioration → best supportive care | 16.10.2020 | Disease progression of Burkitt lymphoma with multisystem involvement Complications related to organ failure in the context of disseminated malignancy and HIV co-infection | Patient presented with systemic B symptoms, bulky lymphadenopathy, and extensive extranodal sites  Patient died within days of diagnosis due to progressive lymphoma |
| 3 | **BL19** | 06.11.2021 | | 62 | IIIB | COVID-19 of moderate severity, with associated pneumonia (U07.2) and severe comorbidities, including ischemic cardiomyopathy, stage 3 hypertension (risk 4), congestive heart failure (NYHA III), bilateral pleural effusion, hypothyroidism, type 2 diabetes (newly diagnosed), anemia of unknown origin | No | Hepatic dysfunction, poor PS | 11.12.2021 | Burkitt lymphoma progression with hepatic dysfunction, heart failure, and COVID-19 pneumonia |  |
| 4 | **BL20** | 17.06.2022 | | 54 | IIB | Chronic liver dysfunction with ascites and impaired hepatic function | No | Severe intoxication, impaired liver function, rapid deterioration | 19.06.2022 | Patient died shortly after diagnosis due to progression of aggressive Burkitt lymphoma with extensive gastric involvement, severe intoxication, liver failure, and multi-organ dysfunction. | Patient became non-transportable to specialized center due to rapid clinical deterioration. Chemotherapy initiation was contraindicated because of impaired liver function. |
| 6 | **BL25** | 10.02.2020 | | 40 | IV | HIV-positive, chronic hepatitis C infection | Yes | Patient refused both chemotherapy and ARV therapy | 20.02.2020 | Lymphoma related (tumor intoxication, multi-organ failure) | The patient presented with disseminated stage IV disease involving multiple organ systems, including the liver, spine, and retroperitoneal lymph nodes. He was severely debilitated at admission, with ECOG 4, signs of tumor-related intoxication, and cachexia. His HIV infection remained untreated. Despite multidisciplinary evaluation, the patient refused both oncologic and antiretroviral therapy. Due to the rapidly progressing disease and poor performance status, treatment could not be initiated. He died 10 days after diagnosis from multi-organ failure. |
| 7 | **BL26** | 29.04.2015 | | 42 | IV | Chronic heart failure with reduced ejection fraction NYHA class III  Type 2 diabetes  Chronic kidney disease, stage 3 | No | Poor PS, multiple comorbidities | 18.07.2015 | Progressive disease is complicated by decompensated heart failure and multi-organ failure in the setting of advanced lymphoma and chronic comorbid conditions. | The patient presented with advanced stage IV lymphoma and significant cardiac and renal impairment. Despite initial consideration, aggressive treatment was contraindicated due to high risk of treatment-related morbidity and mortality. Supportive management focused on symptom control and optimization of comorbid conditions. |
| 8 | **BL42** | 13.05.2016 | | 30 | IIIA | History of recurrent bacterial respiratory infections  Anemia of chronic disease  Chronic obstructive pulmonary disease, moderate severity | No | Poor PS (ECOG 3), comorbidities | 21.06.2016 | Respiratory failure secondary to progressive pulmonary infection on the background of chronic obstructive pulmonary disease and lymphoma progression. | The patient presented with advanced-stage lymphoma and significant comorbidities that limited therapeutic options. Supportive care was prioritized. Despite best efforts, rapid disease progression coupled with recurrent infections led to deterioration and death within 6 weeks of diagnosis. |
| 9 | **BL103** | 07.12.2024 | | 19 | III | No known comorbidities | No | Fulminant course, suspected CNS involvement, GCS 6, family opted for BSC | 09.12.2024 | Disease-related multiorgan failure in the context of advanced-stage lymphoma with suspected CNS involvement. | The patient was diagnosed with stage III aggressive lymphoma on 07.12.2024 and died on 09.12.2024. The disease course was marked by acute neurological decline and signs of systemic decompensation. Due to the patient's critical condition, oncologic treatment was not initiated. Supportive care included hemodynamic stabilization, corticosteroids, and symptom management. |

Abbreviations: ARV: Antiretroviral Therapy; CHF: Congestive Heart Failure; CKD: Chronic Kidney Disease; CNS: Central Nervous System; COPD: Chronic Obstructive Pulmonary Disease; ECOG: Eastern Cooperative Oncology Group; GCS: Glasgow Coma Scale; HIV: Human Immunodeficiency Virus; IVB, IIIB, etc.: Ann Arbor Staging System (Stage and Substage); MDT: Multidisciplinary Team; NYHA: New York Heart Association; PO: Per Os (by mouth); R/R: Relapsed/Refractory; U07.2: ICD-10 Code for COVID-19, virus not identified.

**SUPPLEMENTARY TABLE 2.** Clinical characteristics/differences among the three treatment groups.

| **N = 95** | **R-BFM (n=42)** | **R-EPOCH (n=22)** | **R-CODOX-M / R-IVAC (n=17)** | **R-Hyper CVAD (n=15)** |
| --- | --- | --- | --- | --- |
| **Median age, years (range)** | 8 (2-17) | 58 (26-80) | 44 (19-68) | 37 (18-64) |
| **Male** | 31 | 13 | 9 | 9 |
| **Age> 40 years** | 0 | 11 | 10 | 3 |
| **MYC rearrangement** | 12 | 5 | 1 | 15 |
| **Ann Arbor stage 3-4** | 22 | 16 | 8 | 12 |
| **B symptoms** | 21 | 15 | 9 | 10 |
| **ECOG performance status 2-4** | 20 | 16 | 9 | 12 |
| **>1 extranodal site (n=42 assessed)** | 3 (all CNS) | 1 | 5 | 8 |
| **Elevated LDH** | 19 | 10 | 12 | 4 |
| **EBV positive (N=74 assessed)** | 6 | 6 | 6 | 2 |
| **Bulk >9cm** | 13 | 12 | 8 | 9 |
| **HIV positive** | 0 | 0 | 1 | 0 |
| **CNS involvement** | **4** | **2** | **1** | **0** |
| **BM involvement** | **0** | **0** | **0** | **0** |
| **IPI score 4-5** | **1** | **8** | **3** | **4** |

Abbreviations: BM: Bone Marrow; CNS: Central Nervous System; EBV: Epstein-Barr Virus; ECOG: Eastern Cooperative Oncology Group; IPI: International Prognostic Index; IVAC: Ifosfamide, Etoposide, and high-dose Cytarabine; LDH: Lactate Dehydrogenase; MYC: v-myc avian myelocytomatosis viral oncogene homolog; R-BFM: Rituximab-based Berlin-Frankfurt-Münster protocol; R-CODOX-M: Rituximab, Cyclophosphamide, Vincristine, Doxorubicin, high-dose Methotrexate; R-EPOCH: Rituximab, Etoposide, Prednisone, Vincristine, Cyclophosphamide, Doxorubicin; R-Hyper CVAD: Rituximab, Hyperfractionated Cyclophosphamide, Vincristine, Doxorubicin, Dexamethasone.

**
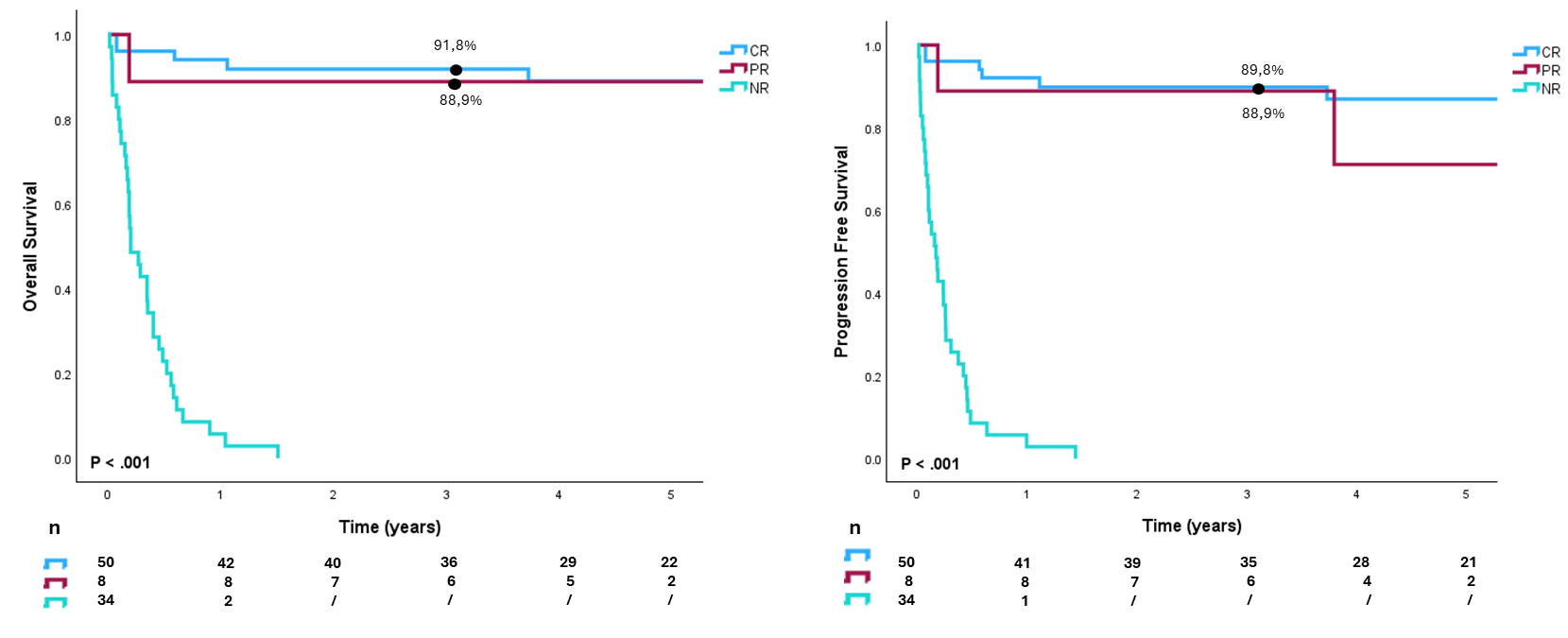
**

**SUPPLEMENTARY FIGURE 1.** Kaplan-Meier survival curves illustrating clinical outcomes based on response to therapy in our cohort. Panel A) Overall Survival (OS). Patients achieving complete remission (CR, blue) or partial remission (PR, red) had significantly better OS at 3 years (92% and 89%, respectively), compared to those with no response (NR, cyan), who experienced poor survival outcomes (P < .001). Panel B) Progression-Free Survival (PFS). Similarly, both CR and PR groups showed favourable PFS over time, while the NR group had significantly inferior PFS, with rapid progression within the first year (P < .001).

**
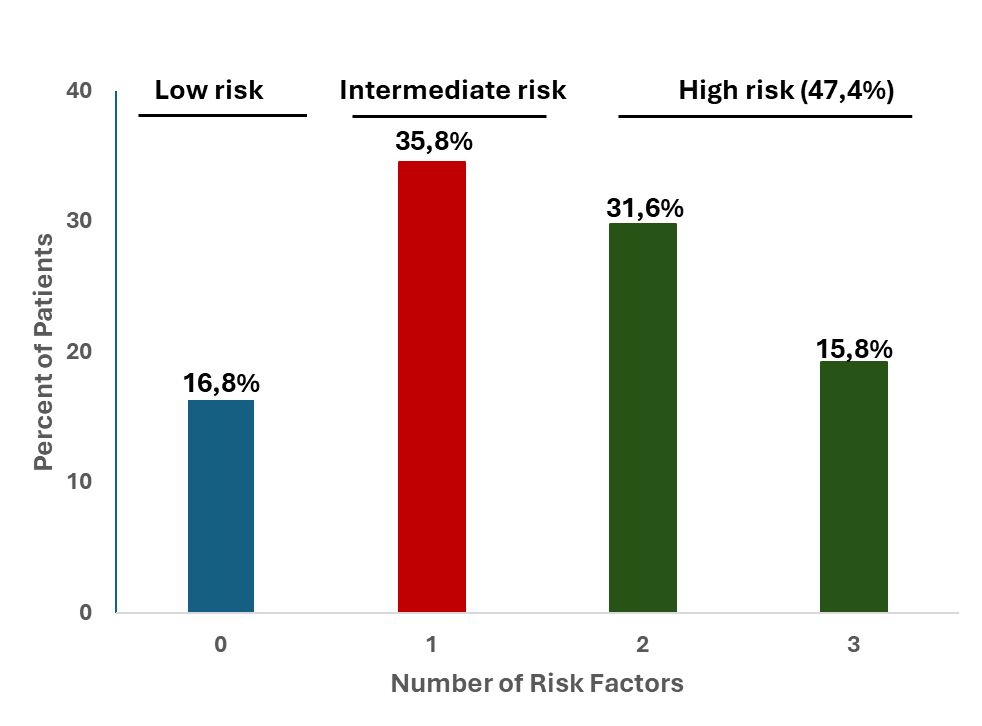
**

**SUPPLEMENTARY FIGURE 2.** Percentage of patients according to the number of risk factors (Age ≥40; Performance status≥ 2; LDH>UNL; CNS involvement).
